# Odor–Taste Interactions in Food Perception: Exposure Protocol Shows No Effects of Associative Learning

**DOI:** 10.1093/chemse/bjab003

**Published:** 2021-01-21

**Authors:** Robin Fondberg, Johan N Lundström, Janina Seubert

**Affiliations:** 1 Division of Psychology, Department of Clinical Neuroscience, Karolinska Institutet, 17177 Stockholm, Sweden; 2 Monell Chemical Senses Center, Philadelphia, PA 19104, USA; 3 Department of Psychology, University of Pennsylvania, Philadelphia, PA 19104, USA; 4 Stockholm University Brain Imaging Centre, Stockholm University, 10691 Stockholm, Sweden

**Keywords:** associative learning, conditioning, flavor, flavor binding, mere-exposure

## Abstract

Repeated exposure can change the perceptual and hedonic features of flavor. Associative learning during which a flavor’s odor component is affected by co-exposure with taste is thought to be central in this process. However, changes can also arise due to exposure to the odor in itself. The aim of this study was to dissociate effects of associative learning from effects of exposure without taste by repeatedly presenting one odor together with sucrose and a second odor alone. Sixty individuals attended two testing sessions separated by a 5-day Exposure Phase during which the stimuli were presented as flavorants in chewing gums that were chewed three times daily. Ratings of odor sweetness, odor pleasantness, odor intensity enhancement by taste, and odor referral to the mouth were collected at both sessions. Consistent with the notion that food preferences are modulated by exposure, odor pleasantness increased between the sessions independently of whether the odor (basil or orange flower) had been presented with or without sucrose. However, we found no evidence of associative learning in any of the tasks. In addition, exploratory equivalence tests suggested that these effects were either absent or insignificant in magnitude. Taken together, our results suggest that the hypothesized effects of associative learning are either smaller than previously thought or highly dependent on the experimental setting. Future studies are needed to evaluate the relative support for these explanations and, if experimental conditions can be identified that reliably produce such effects, to identify factors that regulate the formation of new odor–taste associations.

## Introduction

Past experience helps our perceptual system to integrate sensory information from distinct modalities into a coherent percept with semantic and hedonic features (Lewkowicz and Ghazanfar 2009). For example, frequent exposure to specific odor–taste combinations changes how their combined percept, the food’s flavor, is perceived and evaluated. This plasticity is thought to be central for survival, as it promotes consumption of familiar food items that have been determined through experience to be safe and nutritious. Associative learning within the olfactory and gustatory network has been proposed to account for effects of exposure on flavor perception (Prescott 1999). However, very few features of flavor have been directly manipulated in associative learning paradigms.

Flavors are holistic percepts that arise from inside the mouth during food consumption (Spence 2016). They rely on cortical integration of odor and taste signals that have been processed separately in the peripheral nervous system before reaching the cortex (Boesveldt 2017). Observations from everyday life indicate that repeated exposure to specific odor–taste mixtures during habitual food consumption modulates how the odor is subsequently perceived when presented alone. Most notably, some odors that are often experienced in combination with sweet taste, such as strawberry or vanilla, are commonly described as “smelling sweet” (Dravnieks 1985). In addition to affecting our experiences of unisensory odors, there is reason to believe that exposure to odor–taste mixtures also affects how their sensory components interact when subsequently presented together. Specifically, flavor percepts are perceived and hedonically evaluated based on the extent to which the specific combination of unisensory components resembles a familiar food item (e.g., Schifferstein and Verlegh 1996; Lim and Johnson 2012). Three such phenomena have been frequently described in the literature:

First, the degree of familiarity of an odor–taste mixture affects its hedonic value (Schifferstein and Verlegh 1996; Small et al. 2004; Fondberg et al. 2018). By presenting different odor–taste mixtures in water solutions to the mouth, familiar combinations (e.g., vanilla odor + sweet taste or chicken odor + salty taste) have repeatedly been found to be more palatable than unfamiliar combinations (e.g., vanilla + salty or chicken + sweet).

Second, presenting a taste together with an odor will enhance the intensity of the odor, but only if that specific odor–taste combination has been frequently perceived in the past (Green et al. 2012; Fujimaru and Lim 2013; Lim et al. 2014; Linscott and Lim 2016). For example, adding sucrose to a vanillin solution enhances the perceived intensity of the retronasal vanilla odor, whereas adding salt does not.

The third phenomenon is the olfactory location illusion, often labeled as odor referral, in which the odor component of flavor is perceived as originating from the mouth (Hollingworth and Poffenberger 1917; Rozin 1982; Spence 2016). For example, the vanilla odor of vanilla custard is, just like the sweet taste, experienced in the mouth and not in the nasal cavity where the olfactory receptors are located. This perceptual illusion occurs more frequently when the odor–taste combination is familiar than when it is unfamiliar (Lim and Johnson 2011, 2012; Lim et al. 2014; Fondberg et al. 2018; but see also Stevenson et al. 2011).

Both perceived odor sweetness and the effects of familiarity on flavor perception are typically interpreted within the broader framework of associative learning (Prescott 2012; Stevenson 2014). This idea is based on the realization that consumption of any food item will cause synchronized stimulation with a specific odor–taste pair. When an odor and a taste is repeatedly perceived together, such as vanilla and sweet, an associative link will be formed between the two. The unisensory olfactory and gustatory qualities in combination with the newly formed multisensory associations will then determine the coherent flavor experience of the food item (Stevenson and Oaten 2010). Within this framework, the perceived sweetness of vanilla odor is thought to reflect the strength of its association with sweet taste.

Studies on associative flavor learning do in general include at least two odors. One that is presented together with a taste, and another one that is presented alone (or with a different taste, e.g., Yeomans 2006; Yeomans and Mobini 2006; Stevenson and Mahmut 2011a). Changes in perceptual and hedonic features can then be compared between the odors to isolate effects of associative learning from effects that might have been caused by exposure to the odor alone. Distinguishing between these two learning processes is particularly important when studying pleasantness, as previous work has suggested that the hedonic tone of odors can be influenced both by exposure to pure odors (Delplanque et al. 2015) and by exposure to odors together with sweet taste (Yeomans et al. 2006). While there is no reason to expect that odors will become sweeter due to exposure alone, the inclusion of an odor presented without taste allows one to test if the increase in odor sweetness will be greater for sweet-paired than for unpaired odors. By using this setup, the potential influence of any non-associative effects can thus be controlled (Stevenson et al. 1998).

Such experimental designs are relatively straightforward in theory. However, delivering chemosensory stimuli with the necessary control has been shown to be a non-trivial task. Indeed, to date, learning-dependent aspects of flavor perception have only been studied by a few research groups and results have been mixed. Out of the four above-mentioned phenomena that have been hypothesized to be driven by associative learning, research on odor sweetness has produced the most consistent results. Several studies with various designs have reported that repeated exposure with sucrose can make odors smell sweeter (Stevenson et al. 1995, 1998, 2000a, 2000b; Stevenson and Case 2003; Prescott et al. 2004; Prescott and Murphy 2009; Yeomans et al. 2009; Stevenson and Mahmut 2011a, 2011b; Privitera et al. 2012; Yeomans and Boakes 2016; but see also Sundqvist et al. 2006). For example, in a classic study by Stevenson and colleagues (1998), participants first completed a pretest where two orthonasal odorants were rated in terms of smelled sweetness. The odorants were chosen to be moderately sweet and relatively unfamiliar. On the following 3 days, participants made daily visits to the lab to sample solutions with different flavorants. Three samples always contained one of the target odorants and sucrose, and three always contained the other target odorant without sucrose. After this Exposure Phase, ratings of smelled sweetness were collected once again. A significant increase in orthonasal sweetness was revealed, but only for the odor that had been paired with sweet taste.

Results are less clear for potential pleasantness effects. Some studies indicate that the positive or negative hedonic values of sweet or bitter tastes indeed can be transferred to odors following exposure to odor–taste mixtures (Zellner et al. 1983; Baeyens et al. 1990, 1995, 1996; Stevenson and Case 2003; Mundy et al. 2006; Dickinson and Brown 2007; Wardle et al. 2007; Barkat et al. 2008; Prescott and Murphy 2009; Yeomans et al. 2009; Privitera et al. 2012; van den Bosch et al. 2015; Yeomans and Boakes 2016; Ruszpel and Gast 2020), but other studies have not found any effects on pleasantness (Baeyens et al. 1990; Stevenson et al. 1995, 1998, 2000a, 2000b; Sundqvist et al. 2006; Barkat et al. 2008; Stevenson and Mahmut 2011a, 2011b; van den Bosch et al. 2015). These conflicting results may be explained by interindividual differences in taste preferences. This notion has gained support from a study where odor pleasantness indeed increased more after exposure with sucrose than after exposure without taste, but only for participants that liked sweet taste to begin with (Yeomans 2006). Evidence so far thus indicates that sweet-liking is a necessary prerequisite for associative learning when it comes to odor pleasantness. This means that sweet-paring is expected to only modulate the potential effect of exposure if the consumer likes sweet taste.

Evidence for associative learning effects on odor intensity, that is, the perceived strength of the overall sensation (not only sweetness), is weak. Some studies indicate that unisensory odors are rated as more intense following exposure with taste than after exposure without taste (Stevenson et al. 1995, 1998, 2000a). However, one study has reported an effect the opposite direction (van den Bosch et al. 2015), and several studies did not find any significant effects at all (Stevenson et al. 2000b; Stevenson and Case 2003; Sundqvist et al. 2006; Labbe and Martin 2009; Yeomans et al. 2009; Stevenson and Mahmut 2011b; Yeomans and Boakes 2016). These studies thus provide no conclusive evidence that associative learning influences the intensity of unisensory odors. Because intensity was not the main focus of any of the above-mentioned studies, a thorough discussion about what might have caused these conflicting results is still lacking in the literature. If associative learning does influence the intensity of unisensory odors, more work needs to be devoted to identifying experimental conditions that reliably produce this effect. One closely related question is whether the odor intensity enhancement resulting from presenting a taste together with an odor might increase with exposure. This hypothesis has gained indirect support from studies showing that odor enhancement by taste is larger for familiar than for unfamiliar combination (Green et al. 2012; Fujimaru and Lim 2013; Lim et al. 2014; Linscott and Lim 2016). While this phenomenon seems to be quite robust, no learning study to date has been designed to test if such effects can be induced by exposure. Similarly, potential effects of associative learning on odor referral to the mouth also remain to be determined.

While there is little doubt that experience is important for how we perceive and emotionally respond to foods, it is still not clear to what degree odor–taste interactions rely on the formation of associations between the olfactory and gustatory modalities, how easily such associations are formed, and how strong the potential associative learning effects are in relation to effects of exposure without taste.

This present study has two overall aims. First, to conceptually replicate the previously reported associative learning effects on orthonasal odor sweetness and retronasal odor pleasantness. Second, to directly test whether odor–taste associative learning also affects odor intensity enhancement by taste, and the likelihood of odor referral to the mouth. To separate changes due to associative learning from changes due to exposure without taste, we will compare ratings of odors that have been repeatedly presented with and without sucrose during an extensive Exposure Phase. Specifically, four hypotheses will be tested:

1. Perceived odor sweetness will increase more after exposure with a sweet taste than after exposure without taste (associative learning).
2. Perceived odor pleasantness will increase following exposure independently of whether the odor has been exposed with or without sweet taste (exposure effect without associative learning). The degree to which participants like sweet taste will determine whether exposure with sucrose results in a larger increase than exposure without taste, that is, the increase in pleasantness of sweet-paired odors will be larger than the increase of unpaired odors for participants that like sweet taste (associative learning).
3. Adding a sweet taste to an odor solution will enhance the intensity of the combined solution more if the odor has been previously exposed with sweet taste, than if it has been exposed without taste (associative learning).
4. Odor referral to the oral cavity and tongue will occur more frequently if the odor has been exposed with sweet taste, than if it has been exposed without taste (associative learning).

Hypothesis 1 will be tested using orthonasal odors presented via the nostrils, whereas Hypotheses 2–4 will be tested using retronasal odor solutions presented via the mouth and nasopharynx with (3 and 4) or without (2) sucrose. However, unlike previous work, we will present the flavorants in chewing gums instead of liquid solutions to maximize the amount of exposure.

## Method

### Participants

We decided a priori to terminate data collection when 60 individuals (40 women, *M*_age_ = 27.31, *SD*_age_ = 5.05) had completed both testing sessions. Our sample size was determined pragmatically, balancing a large subject number relative to previous studies with the given resource constrains. One individual did not return for the second session and was therefore not included in the final sample; hence, 61 individuals in total were recruited through an online testing recruitment system hosted by Karolinska Institutet. To be eligible, participants had to be 18–45 years old and speak English. Exclusion criteria were tobacco use, current cold or flu symptoms, self-reported taste or smell dysfunction, and less than 11 out of 16 points on an olfactory screening test where 10 or below indicates olfactory dysfunction (Sniffin’ Sticks; Hummel et al. 1997). All participants provided written informed consent and received a small payment on completion. Participants were asked not to eat or drink flavored beverages 1 h before each testing session to limit potential odor acuity effects. This study conformed to the Declaration of Helsinki and was approved by the Regional Ethics Review Board in Stockholm. Data were collected in the fall-winter of 2019.

### Preregistration and data/material availability

The preregistration, the PsychoPy scripts used to control the experiment and collect responses, the study protocol, the exact randomization procedure, the stimulus recipes, and the analysis scripts have been uploaded to the Open Science Framework (https://osf.io/dtv8s/). Raw data are available on request due to confidentiality restrictions in the ethics protocol.

### Stimuli

#### Odor selection

Two odorants, basil (Basil oil, Stockholms Aeter Essencefabrik AB) and orange flower (Orange flower oil, Stockholms Aeter Essencefabrik AB), were selected from a large set of odorants to be clearly distinguishable from each other, not identifiable, neutrally valenced, and tasteless when dissolved in water.

These odorants were presented both orthonasally (sniffed from bottles when assessing odor sweetness) and retronasally (sipped from medicine cups when assessing the other outcomes) in different concentrations. All odor and taste stimuli were made freshly every 48 h, stored at 6°C in sealed glass containers, and presented at room temperature.

#### Retronasal odorants and tastants

Retronasal odorants were produced by dissolving the odorous oils in 96% ethanol (0.48% volume/volume [v/v]) and then diluting the solvents to target concentrations with tap water. Tap water was chosen instead of bottled water as we judged it to be completely tasteless when mixed with the taste and/or odorants. Moreover, the alcohol was not consciously detectable in any of the stimuli. Concentrations of the retronasal basil (0.0029% v/v) and orange flower (0.0043% v/v) odorants were selected through pilot testing (*n* = 10) to be clearly perceivable but not to evoke any gustatory or trigeminal sensations. To produce combined odor–taste stimuli, sucrose was added to each of the two odorants at a concentration that perceptually mimicked moderately sweet drinks (1.19% weight/volume [w/v]). In addition to the two pure odorants and the two odor–sucrose solutions, participants were also presented with the pure tastant (sucrose and water, 1.19% w/v) and plain water as controls.

The widespread belief that sweetness is universally liked has been challenged by studies demonstrating distinct and highly diverse response patterns across individuals (Kim et al. 2017). To quantify interindividual variability in sweet-liking, an intensely sweet taste solution (34.23% w/v) was prepared by mixing sucrose and water. This concentration has been shown to optimally discriminate between different sweet-liking phenotypes (Iatridi et al. 2019). Finally, a retronasal citrus-like odorant used for practice was prepared by mixing citral (0.0025% v/v) and ethanol (0.2% v/v) with water.

#### Orthonasal odorants

The two orthonasal odorants were prepared by first dissolving the odorous oils in ethanol (0.48% v/v for both basil and orange flower) and subsequently diluting the solutions to target concentrations with water (0.033% v/v). Concentrations were determined through a pilot study (*n* = 10) to match the intensities of the retronasal odorants. An orthonasal citrus-like training odorant was then prepared in a similar fashion by mixing citral (0.1% v/v), ethanol (4.0% v/v), and water.

#### Chewing gums

Basil and orange flower flavored chewing gums were used to expose participants to the odors (with or without sweet taste) between the testing sessions. Four different flavor categories of gum were prepared: pure basil, basil-sucrose, pure orange flower, and orange flower-sucrose.

To prepare the gums, the tasteless and odorless gum base (Glee Gum base, https://www.gleegum.com/) was first heated until the solid grains had melted. One of the odorous oils was then added, after which the gum dough was kneaded for at least 10 min. When the oil was evenly distributed and the dough had a firm consistency, it was cut into small pieces of 1.2 g (±0.2 g). Concentrations were selected to produce intensity matched (assessed in *n* = 10) basil (400 µg/100 g gum base) and orange flower (1250 µg/100 g gum base) chewing gums that evoked distinct olfactory, but neither gustatory nor trigeminal sensations. The sweet chewing gums were made in the same way, but in addition to the odorous oil, sucrose was added at a concentration chosen to match commercially available sweet chewing gums (13.04% w/w).

All chewing gums were wrapped in baking paper and stored in plastic bags for no more than 4 weeks to preserve flavor.

### Procedure

Participants attended two testing sessions of approximately 75 min that were spaced 5–11 days apart (*M* = 6.37, *SD* = 1.57). At the end of the first session, participants were in alternating order either given 15 sweetened basil gums and 15 unsweetened orange flower gums, or 15 unsweetened basil gums and 15 sweetened orange flower gums. In each batch, the chewing gums of each flavor were numbered 1–15.

**Figure 1.**
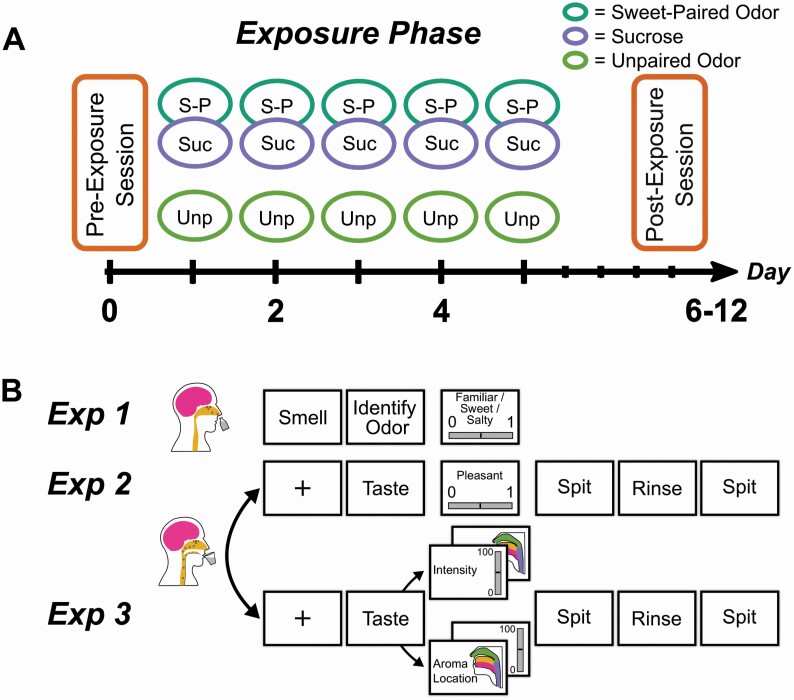
Procedure and trial design. (A) Timeline description of the testing procedure. Ratings were obtained during the pre-exposure and post-exposure sessions. A 5-day Exposure Phase separated the testing sessions during which participants chewed two types of chewing gums: one flavored with an odor and sucrose (the sweet-paired odor), and one flavored with a second odor without sucrose (the unpaired odor). (B) Trial design of the three experiments. Experiment 1 (Exp 1) assessed odor sweetness, Experiment 2 (Exp 2) odor pleasantness, and Experiment 3 (Exp 3) odor intensity enhancement by taste and odor referral to the mouth. To avoid order effects, the sequence of Experiments 2 and 3 was alternated between participants and during Experiment 3, intensity and odor referral (aroma location) were assessed in random order.

During the next 5 days, subsequently referred to as the Exposure Phase, participants chewed six chewing gums per day: two before breakfast, two before lunch, and two before dinner (see Figure 1A). One of the two chewing gums was always sweetened, and the other unsweetened. We emphasized that all gums should be chewed before meals to maximize their salience. Participants were instructed to chew the first gum for 60 s, then pause for 5 min without eating and drinking, and then chew the other gum for 60 s. Feedback from the pilot phase indicated that the flavors were well preserved throughout the first minute of chewing. To avoid order effects, participants alternated between chewing the sweetened or the unsweetened gum first between days. Every night during the Exposure Phase, the participants texted one of the experiment leaders to confirm that all gums had been chewed. To ensure that no gum was skipped, participants were (incorrectly) told that one of the chewing gums could be salty. The instruction was that if they found the salty gum, they should mention the salty gum number in that night’s text message. Participants that failed to send a text message were contacted without delay and reminded of the importance of sticking to the routine. Overall, compliance was high: all 60 participants had contact with the experiment leader according to schedule, or no later than 1 day late, to confirm that all gums had been chewed and that none had been salty.

The pre- and post-exposure sessions each contained three experiments that were separated by short pauses to minimize habituation. Before the experiments began, participants rated the pleasantness of the intensely sweet sucrose solution used to measure sweet-liking. While having 3 mL of the solution in their mouth, the question “How much do you like this taste?” was displayed on the screen, together with a visual analog scale with endpoint anchors “Not at all” and “Very much.” After clicking on the scale, participants expectorated and rinsed thoroughly with water. These ratings were collected at both testing sessions to ensure that potential changes in the outcomes could safely be attributed to learning and not to methodological differences between the sessions. To reduce the risk of spillover effects, the intensely sweet sucrose solution was presented at least 2–3 min before the target odors in the first experiment.

Experiment 1 assessed orthonasal odor sweetness, Experiment 2 retronasal odor pleasantness, and Experiment 3 both retronasal odor intensity enhancement by taste and retronasal odor referral to the mouth (see Figure 1B). To ensure that the intensely sweet sucrose solution would not affect the subsequent retronasal sensations, all participants first completed Experiment 1 that only contained orthonasal stimuli. Then, to avoid order effects, the sequence of Experiments 2 and 3 was alternated between participants. Just before the beginning of each experiment, the upcoming task was explained in detail. Then, a practice trial with the citrus-like orthonasal (Experiment 1) or retronasal (Experiments 2 and 3) training odorant was used to ensure that the participant understood the task and felt comfortable using the scales.

By presenting all retronasal stimuli in medicine cups, participants were briefly exposed to the orthonasal odor components in the solutions before they perceived them retronasally in the mouth. This setup mimics normal eating conditions, where food odors always reach the nose by the orthonasal route first.

#### Experiment 1: Odor sweetness

This experiment assessed changes in orthonasal odor sweetness between the testing sessions. We only expected the perceived sweetness to increase for odors that had been paired with sucrose during the Exposure Phase (Hypothesis 1).

After the practice trial, the basil and orange flower odorants were presented and rated one at a time. For each odorant, participants were instructed to open a bottle and sniff while placing the opening 2–3 cm below the nose. The first question to appear on the screen was “What does it smell like?” along with a textbox field for participants to provide a free form response. Next, three visual analog rating scales appeared on the same frame, each presented as a horizontal line placed across the top, middle, or low section of the screen, respectively. The scales measured familiarity, sweetness, and saltiness, in that order, and used “Not familiar/sweet/salty at all” and “Extremely familiar/sweet/salty” as endpoint anchors. Saltiness ratings were collected as a control to ensure that the potential effect of the Exposure Phase was taste-specific (this secondary analysis are presented in the “Supplementary results”-folder on https://osf.io/dtv8s/) and quality + familiarity ratings were collected to ensure that the odors were not identifiable at the first testing session. Clicking on a scale displayed a marker that could be dragged along the horizontal axis. Participants were allowed to smell the odorants as many times as necessary. This experiment ended when all three ratings had been completed. The exact interstimulus intervals depended on how much time the participants spent on each question, but most trials lasted a couple of minutes.

#### Experiment 2: Odor pleasantness

This experiment assessed changes in retronasal odor pleasantness between the sessions and tested if the degree to which participants liked sweet taste modulated the potential amplifying effect of sweet-pairing (Hypothesis 2).

The two pure odorants, the two odorant-sucrose mixtures, the pure tastant, and plain water were presented twice in one out of four pseudo-randomized presentation orders (without repetitions). In total, this experiment thus contained 12 trials. Participants evaluated each stimulus by tasting a 3 mL sample from a numbered medicine cup, and swishing the solution around in the mouth while breathing through the nose. While our preregistered hypotheses only focused on the pure odorants, we also included odorant-sucrose mixtures in this stimulus set to allow for exploratory follow-up analyses. If an associative learning effect on odor pleasantness had emerged, we would have tested the generalizability of the effect by also analyzing flavor pleasantness.

All trials had the same structure. They were initiated by a fixation cross (3 s), after which “Please taste cup no. X” appeared on the screen (6.5 s). While the participant had the stimulus solution in the mouth, the question “How much do you like this taste?” was displayed together with a visual analog scale with endpoint anchors “Not at all” and “Very much.” Participants rated their hedonic experience by clicking on the scale. They were then instructed to expectorate the solutions (5.5 s), rinse with water (7.5 s), and expectorate again (5.5 s).

#### Experiment 3: Odor intensity enhancement by taste and odor referral to the mouth

This experiment tested two hypotheses (3 and 4): first, if changes between the sessions in odor intensity enhancement by taste were amplified by sweet-pairing. Specifically, we expected that adding sucrose to an odor solution would result in a larger increase in perceived intensity at the second session than at the first session, but only for odors that had been paired with sucrose during the Exposure Phase. Second, if odor referral to the oral cavity and tongue increased between the sessions. Again, we only expected increased referral for odors that had been paired with sucrose during the Exposure Phase.

The same stimulus material and presentation order were used as in Experiment 2. Each trial began with a fixation cross (3 s) that was followed by a prompt (7 s) to taste the solution in one of the 12 numbered medicine cups. Two tasks were then presented consecutively in random order with a short pause (0.5 s) in between. One task required the participants to localize the sensation evoked by the solution’s olfactory (aroma) component, while trying to ignore any taste sensations. To ensure correct task performance, participants were trained to separate the taste (“the sweet, salty, bitter, sour, or umami part of a flavor”) from the odor/aroma (“any other flavor-like sensation”) component of flavor just before the experiment began. For each trial, the question “Where do you perceive the aroma?” was displayed on the screen together with a cross-sectional illustration of a human head (adapted from Lim and Johnson 2012) with the anatomical locations “Nose,” “Oral cavity,” “Tongue,” and “Throat.” Participants could select none, one, or several of the locations to indicate where they perceived the odor component. The other task required participants to attend to the full flavor experience. “Rate the intensity of this beverage” was displayed on the screen, together with a labeled magnitude scale with the anchors “Barely detectable” and “Strongest imaginable.” Clicking on the scale logged the response and immediately removed the image. After having completed both tasks with the solution in the mouth, participants were instructed to expectorate (5.5 s), rinse with water (7.5 s), and expectorate again (5.5 s).

### Statistical analyses and data transformation

Unless stated otherwise, analyses were carried out in accordance with the preregistered analysis plan.

#### Predictor variables

##### Session.

Mean centered variable indicating whether the rating was collected at the “pre-exposure” (−0.5) or “post-exposure” (0.5) session.

##### Condition.

Mean centered variable indicating whether the rated stimulus’s odor component was presented with (“sweet-paired odor”: 0.5) or without (“unpaired odor”: −0.5), sucrose during the Exposure Phase.

##### Sweet-liking.

Mean centered variable containing each participant’s average pleasantness rating of the intensely sweet sucrose solution across the two sessions. The original ratings were on a scale from 1 (“Not pleasant at all”) to 100 (“Very pleasant”).

##### Days between sessions.

Numeric variable indicating the number of days that had passed between the two testing sessions for each participant.

#### Outcome variables

##### Odor sweetness.

Orthonasal odor sweetness was denoted as a number between 0.01 (“Not sweet at all”) and 1 (“Extremely sweet”), which corresponded to the selected location on the rating scale.

##### Odor pleasantness.

Retronasal odor pleasantness was also denoted as a number between 0.01 (“Not pleasant at all”) and 1 (“Very pleasant”), which again corresponded to the selected location on the rating scale.

##### Odor intensity enhancement (by taste).

A difference score was defined to reflect the difference in intensity between a pure retronasal odor, and the same retronasal odor presented with sweet taste. This variable was obtained by subtracting the intensity rating for each odor solution from the respective intensity rating for the same odor solution when it also contained sucrose. Basil, basil + sucrose, orange flower, and orange flower + sucrose were each presented twice every session, which resulted in four unique difference scores (e.g., intensity [basil + sucrose] – intensity [basil]). The raw intensity ratings were on a scale from 1 (“Barely detectable”) to 100 (“Strongest imaginable”).

##### Odor referral to the oral cavity and tongue.

Two separate binary localization indicators were used to specify whether or not the retronasal odor components of the odor + sucrose solutions had been perceived in the oral cavity (“perceived in the oral cavity”: 1, “not perceived in the oral cavity”: 0) or tongue (“perceived on the tongue”: 1, “not perceived on the tongue”: 0).

### Analyses

Mixed-effects models were used to estimate the effects of Session (“pre-exposure,” “post-exposure”), Condition (“sweet-paired,” “unpaired”), and their interaction. We used linear mixed-effects models (LMM) to analyze odor sweetness, odor pleasantness, and odor intensity enhancement by taste, and generalized mixed-effects models (GLMM) with binomial error distributions and logit link functions to analyze odor referral to the oral cavity and tongue.

Model selection began with the maximal random effects structure justified by the design (Barr et al. 2013). If a model failed to converge, we first increased the number of iterations and tried different numerical optimization procedures (Brauer and Curtin 2018). If the model still did not converge, by-participant random slopes were removed one-by-one, keeping the slope for the effect that was the focus of the confirmatory test for last.

Visual inspection of residual plots (Winter 2013) indicated that the assumptions of homoscedasticity and normality were not met for odor sweetness due to a high proportion of very low ratings. Hence, to check the robustness of the results of the preregistered gaussian models, we also analyzed the sweetness data using mixed-effects beta regression (MEBR). This method works well for bounded data with high skewness (Smithson and Verkuilen 2006). As beta regression is restricted to variables bounded at but not including 1, the single rating of 1 in the sweetness data was replaced with a 0.99. No other obvious deviations from homoscedasticity or normality were observed.

For odor sweetness, odor intensity enhancement, and odor referral, the main hypothesis was that the effect of session would be stronger on sweet-paired odors than on unpaired odors, which would be indicated by a significant interaction term.

To evaluate the sensitivity of the model that assessed odor sweetness, Monte Carlo simulations were conducted using the SIMR package (Green and MacLeod 2016). This exploratory approach allowed us to estimate the power of the specified model to detect different effect sizes. We specified nine evenly distributed effects between 0 (e.g., unpaired odors: no increase in sweetness between the sessions; sweet-paired odors: no increase in sweetness between the sessions) and 0.2 (e.g., unpaired odors: no increase in sweetness between the sessions, sweet-paired odors: a 0.2 [20% of rating scale] increase in sweetness between the sessions). The computed powers with 95% confidence intervals were obtained by using 5000 simulated experiments per effect size. One last exploratory analysis was then conducted to assess whether the Session × Condition interaction varied depending on the number of days that had passed between the two testing sessions. This was done by adding the three-way Session × Condition × “Days between sessions” to the model that had been used to test the Session × Condition interaction.

For odor pleasantness, we had two sub-hypotheses. First, that the pleasantness ratings would increase between the sessions independently of whether the odor had been exposed with or without sucrose during the Exposure Phase. The effect of session was thus expected to be significant (assessed by a model without the interaction term). To compare this effect to potential changes that could have occurred independently of the Exposure Phase, we then used an exploratory paired *t*-test to assess changes in pleasantness (post-exposure ratings vs. pre-exposure ratings) of the retronasal training odorant that only had been presented during the testing sessions.

The second sub-hypothesis related to odor pleasantness was that the two-way Session × Condition interaction would vary depending on how much participants liked sweet taste. As previous studies have indicated that odor pleasantness only increases after exposure with sucrose for participants that like sweet taste, we expected a significant Sweet-liking × Session × Condition three-way interaction, but no Session × Condition two-way interaction. Moreover, to be able to compare our results to previous findings, an exploratory Pearson correlation analysis was then used to test if changes in pleasantness for sweet-paired odors correlated with sweet-liking.

Exploratory equivalence testing was then used to further investigate the non-significant results from the linear models (Lakens et al. 2018). Two one-sided tests assessed if the true effects were smaller than what we considered to be the smallest effect size of interest. This procedure follows the same logic as null hypothesis significance testing, but instead of testing against zero, equivalence tests are designed to determine whether effects that are large enough to be considered meaningful can be statistically rejected. Because a large number of studies have found positive effects on odor sweetness, the smallest effect size of interest for this outcome was determined based on reported effect sizes in the literature (Stevenson et al. 1995, 1998, 2000a, 2000b; Stevenson and Case 2003; Prescott et al. 2004; Prescott and Murphy 2009; Yeomans et al. 2009; Stevenson and Mahmut 2011a, 2011b; Privitera et al. 2012; Yeomans and Boakes 2016). For the other outcomes where the evidence is less consistent, we decided in advance to test against the effect size that a study with 60 participants has 80% power to detect.

All tests were two-tailed, and the alpha level was set a priori at 0.05. *P* values were obtained by likelihood ratio tests comparing models with and without the effects of interest.

We used R (R Core Team, 3.6.3) and R Studio (RStudio Team, 1.2.5033) for all analyses. The statistical packages lme4 (Bates et al. 2015) and glmmTMB (Brooks et al. 2017) were used to fit the mixed-effects models, and TOSTER (Lakens 2017) was used for equivalence testing. Plots were created in ggplot2 (Wickham 2016) and ggstatsplot (Patil 2018).

## Results

Baseline ratings of the orthonasal basil and orange flower odorants obtained at the very beginning of the pre-exposure sessions are summarized in Table 1. Descriptive statistics for each outcome variable are displayed in Table 2 and model equations are reported in Table 3. Non-preregistered analyses are labeled as exploratory. Detailed descriptions of the models and the model selection process are provided in the “Supplementary results”-folder on https://osf.io/dtv8s/ together with the secondary analyses specified in the preregistration

**Table 1.** Baseline ratings of the orthonasal odorants

Odor quality	Familiarity— *M*(*SD*)	Sweetness— *M*(*SD*)	Saltiness— *M*(*SD*)
Basil	0.454 (0.277)	0.393 (0.235)	0.378 (0.265)
Orange flower	0.551 (0.283)	0.460 (0.267)	0.214 (0.208)

M, mean, SD, standard deviation. Each point estimate is based on 60 ratings. Odor familiarity, odor sweetness, and odor saltiness were assessed by using three separate scales with “not familiar/sweet/salty at all” (0.01) and “extremely familiar/sweet/salty” (1) as endpoint anchors.

**Table 2.** Summary statistics of all outcome variables

	Pre-exposure			Post-exposure		
	Mean (Median)	*SD*	*N*	Mean (Median)	*SD*	*N*
Odor sweetness						
Unpaired odors	0.408 (0.465)	0.253	60	0.400 (0.430)	0.245	60
Sweet-paired odors	0.445 (0.485)	0.253	60	0.481 (0.505)	0.244	60
Odor pleasantness						
Unpaired odors	0.391 (0.392)	0.191	120	0.420 (0.433)	0.200	120
Sweet-paired odors	0.404 (0.410)	0.203	120	0.460 (0.473)	0.173	120
Odor intensity enhancement						
Unpaired odors	2.843 (1.722)	13.516	120	2.092 (2.257)	12.357	120
Sweet-paired odors	3.388 (1.377)	15.728	120	3.822 (2.431)	11.150	120
	Pre-exposure			Post-exposure		
Odor referral	Count		*N*	Count		*N*
Referral to oral cavity						
Unpaired odors (*n* = 120)	89		120	99		120
Sweet-paired odors (*n* = 120)	91		120	91		120
Referral to tongue						
Unpaired odors (*n* = 120)	61		120	58		120
Sweet-paired odors (*n* = 120)	56		120	62		120

SD, standard deviation; *N*, total number of observations; Count, number of trials where the odor was localized to the oral cavity or tongue.

**Table 3.** Model equations corresponding to each primary hypothesis

Model name (model type)	Equation
Sweetness1 (LMM)	$Sweetness\;=\;\beta_{0}\;+\;\beta_{1}\;\times \;Session\;+\beta_{2}\;\times \;Condition+\;\beta_{3}\;\times Session\;\times \;Condition\;+\;\mu_{0}\;+\;\mu_{1}\;\times$ Session × Condition + ε
Sweetness2 (LMM)	$Sweetness\;=\;\beta_{0}\;+\;\beta_{1}\;\times \;Session+\beta_{2}\;\times \;Condition+\mu_{0}\;+\;\mu_{1}\;\times \;Session\;+\;\mu_{2}\;\times \;Condition\;+\;\varepsilon$
Pleasantness1 (LMM)	$Pleasantness\;=\;\beta_{0}+\;\beta_{1}\times Session+\beta_{2}\times Condition\;+\;\mu_{0}\;+\;\mu_{1}\;\times \;Session\;+\;\mu_{2}\;\times \;Condition\;+\;\mu_{3}\;\times$ Session × Condition + ε
Pleasantness2 (LMM)	$Pleasantness\;=\;\beta_{0}\;+\;\beta_{1}\;\times \;Session\;\;\;\;+\;\beta_{2}\;\times \;Condition\;\;\;\;+\;\beta_{3}\;\times \;Session\;\times \;Condition\;+\;\mu_{0}\;+\;\mu_{1}\;\times \;Session\;+\;\mu_{2}\;\times \;Condition\;+\;\mu_{3}\;\times \;Session\;\times$ Session × Condition + ε
Pleasantness3 (LMM)	$Pleasantness\;=\;\beta_{0}\;+\;\beta_{1}\;\times \;Session\;\;\;\;+\;\beta_{2}\;\times \;Condition\;\;\;\;+\;\beta_{3}\;\times \;SweetPreference\;+\;\beta_{4}\;\times \;Session\;\times$ $Condition\;+\;\beta_{5}\times \;Session\;\times \;SweetPreference\;+\;\beta_{6}\;\times \;Condition\times SweetPreference+\;\beta_{7}\;\times \;Session\;\times$ $Condition\times SweetPreference+\mu_{0}+\mu_{1}\times Session+\mu_{2}\times Condition+\mu_{3}\times Session\times Condition+\varepsilon$
Intensity1 (LMM)	$Intensity\;Enhancement\;\;\;\;=\;\beta_{0}\;+\;\beta_{1}\;\times \;Session\;\;\;\;+\;\beta_{2}\;\times \;Condition\;+\;\beta_{3}\;\times \;Session\;\times \;Condition\;+$ $\mu_{0}\;+\;\mu_{1}\;\times \;Session\;\times \;Condition\;+\;\varepsilon$
Intensity2 (LMM)	$Intensity\;Enhancement\;=\beta_{0}\;+\beta_{1}\;\times Session+\beta_{2}\times Condition+\mu_{0}+\mu_{1}\times Session\;+\mu_{2}\times Condition+\varepsilon$
OdorReferral_OC1 (GLMM)	$Oral\;Cavity,\;\;\;\;Logit\;\left(Odds\right)\;=\;\beta_{0}\;+\;\beta_{1}\;\times \;Session\;+\;\beta_{2}\;\times \;Condition\;+\;\beta_{3}\;\times \;Session\;\times \;Condition\;+$ $\mu_{0}\;+\;\mu_{1}\;\times \;Session\;\times \;Condition\;+\;\varepsilon$
OdorReferral_T1 (GLMM)	$Tongue,\;Logit\;(Odds)\;=\;\beta_{0}\;+\;\beta_{1}\;\times \;Session\;+\;\beta_{2}\;\times \;Condition\;\;\;\;+\;\beta_{3}\;\times \;Session\;\times \;Condition\;+$ $\mu_{0}\;+\;\mu_{1}\;\times \;Session\;\times \;Condition\;+\;\varepsilon$
OdorReferral_OC2 (GLMM)	$Oral\;\;\;\;Cavity,\;Logit\;\left(Odds\right)\;=\;\beta_{0}\;+\;\beta_{1}\;\times \;Session\;\;\;\;+\;\beta_{2}\;\times \;Condition\;+\;\mu_{0}\;+\;\mu_{1}\;\times \;Session\;+\;\varepsilon$
OdorReferral_OC3 (GLMM)	$Oral\;Cavity,\;Logit\;\left(Odds\right)\;=\;\beta_{0}\;+\;\beta_{1}\;\times \;Session\;\;\;\;+\;\beta_{2}\;\times \;Condition\;+\;\mu_{0}\;+\;\mu_{1}\;\times \;Condition\;+\;\varepsilon$
OdorReferral_T2 (GLMM)	$Tongue,Logit(Odds)=\beta_{0}+\beta_{1}\;\times \;Session\;+\;\beta_{2}\times Condition+\;\mu_{0}\;+\;\mu_{1}\times Session\;+\;\mu_{2}\;\times \;Condition\;+\;\varepsilon$

LMM, linear mixed-effects model; GLMM, generalized mixed-effects model with binomial error distribution and a logit link function, β, fixed effects; μ, random effects, ε, residuals.

### Hypothesis 1. Orthonasal odor sweetness (Figure 2A)

We first tested whether perceived odor sweetness increased more between the testing sessions after exposure with a sweet taste than after exposure without taste. Contrary to expectation, we found no such effect (“Sweetness1” in Table 3, Session × Condition: *b* = 0.044, 95% CI = [−0.045, 0.132], χ ^2^(1) = 0.935, *P* = 0.334). Two exploratory analyses then assessed the robustness of this result. First, the (non-significant) effect did not vary significantly depending on the number of days that had passed between the pre-exposure and post-exposure sessions (χ ^2^(1) = 0.562, *P* = 0.454). Second, the non-Gaussian MEBR provided very similar result (χ ^2^(1) = 0.963, *P* = 0.326). Taken together, these analyses provided no evidence that the sweetness of sweet-paired odors increased more than the sweetness of unpaired odors.

**Figure 2.**
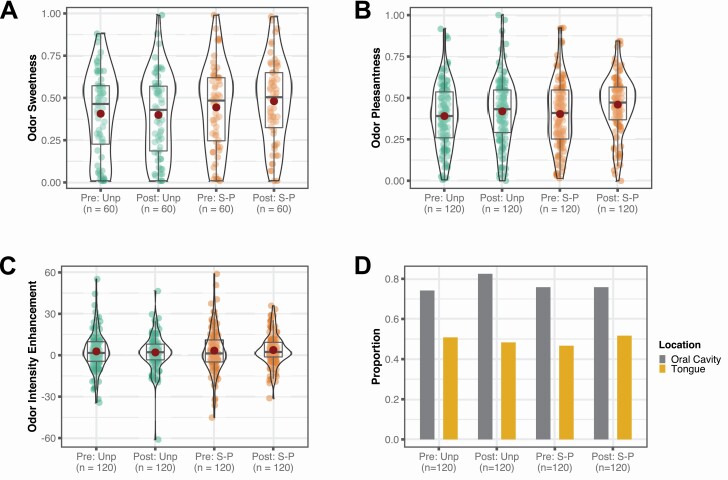
This figure displays the distribution of the outcome variables within each Session (“Pre”-exposure vs. “Post”-exposure) by Condition (“Unp” = unpaired odor vs. “S-P” = sweet-paired odor) context. n is the number of observations per context. In boxplots A–C, the central horizontal lines represent medians and the dark red points represent means. (A) Perceived orthonasal odor sweetness, 0.01 = “Not sweet at all,” 1 = “Extremely sweet.” (B) Perceived retronasal odor pleasantness, 0.01 = “Not pleasant at all,” 1 = “Very pleasant.” (C) Odor intensity enhancement by taste. This score was obtained by subtracting the intensity rating of each odor solution, for example, a solution with only the basil odorant, from the respective intensity rating of the same odor solution when it also contained sucrose, for example, a solution with both basil odorant and sucrose. The raw intensity ratings were on a scale from 1 = “Barely detectable” to 100 = “Strongest imaginable.” (D) Odor referral to the mouth. Proportion of trials where the participants localized the odor component of the odor–sucrose solutions to the oral cavity and tongue, respectively.

**Figure 3.**
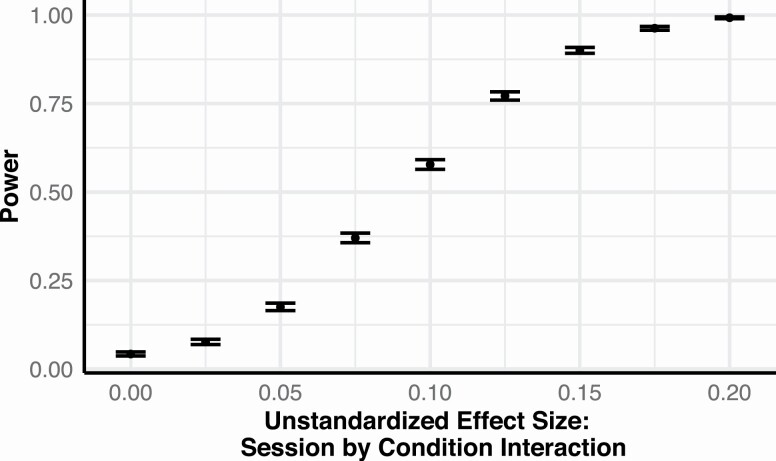
Sensitivity power analyses for Hypothesis 1: Orthonasal odor sweetness. Achieved power for different hypothetical strengths of the Session × Condition interaction (“Sweetness1” in Table 3) that tested if perceived odor sweetness increased more between the testing sessions after exposure with a sweet taste than after exposure without taste. For example, an unstandardized effect of 0.1 could correspond to a 0.10 (10% of the rating scale) increase in sweetness of the sweet-paired odor and no increase of the unpaired odor. Error bars depict 95% confidence intervals based on 5000 simulations. α = 0.05, *n* = 60 (number of participants).

This contrasts with previous studies that have reported large changes in odor sweetness after exposure with sucrose, typically corresponding to a 10–33% increase of the total rating scale. We therefore used an explorative sensitivity analysis to assess the statistical power to detect different hypothetical effect sizes. The smallest Session × Condition interaction that this model could detect at 80% power was approximately 0.13 (e.g., a 0.13 [13% of the rating scale] increase in odor sweetness of the sweet-paired odors, and no increase of the unpaired odors), see Figure 3.

Then, to determine whether our results deviated significantly from prior findings, we used an equivalence test to compare our observed change (sweetness of sweet-paired odors at the post-exposure session – sweetness of sweet-paired odors at pre-exposure session) against an equivalence bound of ±0.1 (or ±10% of our rating scale, which corresponded to a Cohen’s *d* of ~0.5). This exploratory test was significant (mean diff. = 0.036, 90% CI [−0.007, 0.079]; upper bound—*t*(59) = −2.471, *P* = 0.008; lower bound—*t*(59) = 5.291, *P* < 0.001), indicating that the effect of sweet-pairing on perceived odor sweetness was either absent or significantly smaller than in previous studies.

We then assessed main effects in a model without the interaction term. This analysis did not reveal any significant changes between the pre-exposure and post-exposure sessions (“Sweetness2” in Table 3, Session: *b* = 0.015, 95% CI = [−0.023, 0.052], χ ^2^(1) = 0.599, *P* = 0.439), or between sweet-paired and unpaired odors (Condition: *b* = 0.059, 95% CI = [−0.000, 0.118], χ ^2^(1) = 3.817, *P* = 0.051). The results were similar in the exploratory MEBR analysis used to check robustness (Session: χ ^2^(1) = 1.210, *P* = 0.271; Condition: χ ^2^(1) = 4.747, *P* = 0.029) but here, the effect of Condition reached significance. This reflects that in our sample, participants rated the sweet-paired odors as slightly sweeter than the unpaired odors across both testing sessions. This difference was, however, small in comparison to the variability within these measures. Importantly, as shown in Figure 2A, the rating distributions were centered in the middle of the scale and no ceiling effects were present. This means that within both conditions, a potential increase in odor sweetness between the sessions would have been reflected in the actual ratings. Hence, the observed difference in sweetness between sweet-paired and unpaired odors likely did not impair our ability to detect any effects of interest.

### Hypothesis 2. Retronasal odor pleasantness (Figure 2B)

We then tested if pleasantness ratings increased between the sessions, independently of whether the odor had been exposed with or without sucrose during the Exposure Phase. A significant main effect of Session supported this hypothesis (“Pleasantness1” in Table 3, *b* = 0.039, 95% CI = [0.009, 0.070], χ ^2^(1) = 6.244, *P* = 0.012). As expected, this model provided no evidence that pleasantness differed between the sweet-paired and unpaired odors (Condition: *b* = 0.034, 95% CI = [−0.003, 0.070], χ ^2^(1) = 3.343, *P* = 0.067).

The significant increase between the sessions could have been due to the repeated presentations during the Exposure Phase, or to effects associated with multiple testing. To explore the latter possibility, we then used an exploratory paired *t*-test to compare ratings (pre-exposure vs. post-exposure) of the retronasal training odor that only had been presented during the testing sessions. The difference was not significant (mean diff. = 0.030, 95% CI = [−0.001, 0.060], *t*(59) = 1.953, *P* = 0.056), but the observed increase was similar in magnitude to the increase among the target basil and orange flower odorants.

In line with our hypothesis, a subsequent model (“Pleasantness2” in Table 3) that also included the interaction term did not support the notion that changes in pleasantness varied depending on whether the odor had been presented with or without sweet taste during the Exposure Phase (Session × Condition: *b* = 0.028, 95% CI = [−0.016, 0.072], χ ^2^(1) = 1.598, *P* = 0.206). The reason why we did not expect the increase between the sessions to vary between sweet-paired and unpaired odors is that not all people like sweet taste. Instead, we hypothesized that the (non-significant) interaction would be modulated by the degree to which participants liked sweet taste. A model that also included a Session × Condition × Sweet-liking interaction did not, however, support this notion (“Pleasantness3” in Table 3, Session × Condition × Sweet-liking: *b* = −0.001, 95% CI = [−0.003, 0.002], χ ^2^(1) = 0.291, *P* = 0.589). To further explore the role of sweet-liking in the development of odor pleasantness, an exploratory Pearson correlation analysis was used to test if changes in pleasantness *for sweet-paired odors* correlated with sweet-liking. To do so, ratings of identical stimuli within each session were first averaged (within participants). We then calculated changes in pleasantness for sweet-paired odors by subtracting pre-exposure scores from post-exposure scores. Next, these difference scores were correlated with sweet-liking to test if changes in liking of sweet-paired odors were linearly associated with the degree to which participants liked sweet taste. In line with our previous result, the correlation coefficient was not significant (*r*(58) = −0.153, 95% CI = [−0.391, 0.105], *P* = 0.245). Last, results from an exploratory equivalence test showed that the correlation was indeed significantly smaller than the upper equivalence bound of *r* = +0.353 (90% CI [−0.355, 0.064], *P* < 0.001), but not significantly bigger than −0.353 (*P* = 0.053). Taken together, these results suggest that given our experimental design, the potential positive correlation between sweet-liking and changes in pleasantness was, at best, weak.

### Hypothesis 3. Retronasal odor intensity enhancement (Figure 2C)

We then tested if changes between the sessions in odor intensity enhancement by taste differed between sweet-paired and unpaired odors. The non-significant Session × Condition interaction did not support this notion (“Intensity1” in Table 3, *b* = 1.185, 95% CI = [−3.215, 5.584], χ ^2^(1) = 0.281, *P* = 0.596). An exploratory equivalence test was then used to assess if changes in odor intensity enhancement between the sessions for sweet-paired odors were reliably smaller than ±*d* = 0.368 (=±3.682 on the original 1:100 scale). Both tests were significant (mean diff. = 0.434, 90% CI = [−1.726, 2.594]; upper bound—*t*(59) = −2.513, *P* = 0.007; lower bound—*t*(59) = 3.184, *P* = 0.001), suggesting that medium-sized effects could be statistically rejected.

As expected, the next model (“Intensity2” in Table 3) that did not include the interaction term provided no evidence that odor intensity enhancement differed between the pre-exposure and post-exposure sessions (Session: *b* = −0.159, 95% CI = [−2.282, 1.965], χ ^2^(1) = 0.022, *P* = 0.883), or between the sweet-paired and unpaired odors (Condition: *b* = 1.138, 95% CI = [−1.428, 3.703], χ ^2^(1) = 0.775, *P* = 0.379).

### Hypothesis 4. Odor referral to the oral cavity and tongue (Figure 2D)

Finally, two models (“OdorReferral_OC1” and “OdorReferral_T1” in Table 3) were used to test if changes in odor referral to any of the anatomical location in the mouth differed between sweet-paired and unpaired odors. Our results did not support this notion as both Session × Condition interactions were non-significant (Oral cavity: *b* = −0.611, 95% CI = [−2.048, 0.744], χ ^2^(1) = 0.821, *P* = 0.365; Tongue: *b* = 0.428, 95% CI = [−0.440, 1.305], χ ^2^(1) = 0.940, *P* = 0.332).

As expected, odor referral to the mouth did not vary significantly between the sessions (oral cavity [“OdorReferral_OC2” in Table 3]: *b* = 0.238, 95% CI = [−0.457, 0.947], χ ^2^(1) = 0.487, *P* = 0.485; tongue [“OdorReferral_T2” in Table 3]: *b* = 0.083, 95% CI = [−0.483, 0.658], χ ^2^(1) = 0.087, *P* = 0.768) or between and the sweet-paired and the unpaired odors (oral cavity [“OdorReferral_OC3” in Table 3]: *b* = −0.276, 95% CI = [−0.990, 0.375], χ ^2^(1) = 0.710, *P* = 0.399; tongue [“OdorReferral_T2” in Table 3]: *b* = −0.030, 95% CI = [−0.506, 0.442], χ ^2^(1) = 0.016, *P* = 0.899).

## Discussion

The aim of this study was to explore the potential role of associative learning in naturalistic odor–taste interactions. We used several outcome measures to cover different aspects of the food selection and ingestion process, all of which have previously been suggested to be modulated by learning. Although our exposure protocol was extensive and the sample size large compared with most previous studies that have reported significant effects (e.g., Stevenson et al. 1995, 1998, 2000a, 2000b; Stevenson and Mahmut 2011a, 2011b), we found no evidence of associative learning effects on any of the outcomes. Moreover, the presence of medium-sized effects was statistically rejected through exploratory equivalence tests, indicating that with the described method, effects of associative learning within the olfactory–gustatory network are either absent or small in magnitude.

In line with our hypothesis, both target odorants were rated as more pleasant at the post-exposure session than at the pre-exposure session. This increase might be explained by the mere-exposure effect, the psychological phenomenon describing the tendency for humans to develop preferences for things that are familiar. That mere-exposure plays a role in the development of odor preferences has been suggested by previous studies that found positive correlations between pleasantness and familiarity (e.g., Distel et al. 1999). This idea also fits well into the broader learning literature where mere-exposure effects have been documented for many types of auditory and visual stimuli (e.g., Montoya et al. 2017). It is important to note, however, that a numerically similar increase also was observed for the citrus-like training odor, which was only presented during the testing sessions and not during the Exposure Phase. As such, we cannot conclusively attribute the increased pleasantness of the two target odors to the repeated exposures between the sessions. It is, however, possible that the single exposure at baseline was sufficient to trigger a mere-exposure effect in all three odorants. Apparent effects on odor sweetness have previously been reported in flavor learning paradigms that contained only two exposure sessions (Stevenson et al. 2000a). One argument against this explanation is that the training odorant was, unlike the basil and orange flower odorants, easy to identify. Most participants correctly labeled it as “citrus,” “lemon,” or “lemon candy.” While there is no experimental evidence that odors need to be novel in order to be affected by exposure, one can speculate that it might take more than a single exposure to alter the hedonic dimension of odors that have been so firmly encoded in memory that they are identifiable even without verbal cues. In line with this reasoning, previous studies on flavor learning have, just like the present study, selected target odors that are not highly familiar (e.g., Stevenson et al. 1995). Follow-up studies are needed to further explore mere-exposure effects on odor pleasantness and the potential role of novelty in this process.

We found no evidence that the observed increase in pleasantness was amplified by sweet-pairing across all participants. This was expected given, as outlined in the introduction, the heterogeneity of results from previous studies. While the notion that sweet-pairing might have amplifying effects on odor pleasantness is firmly grounded in learning theory and rests on the principles of Pavlovian conditioning (De Houwer et al. 2001), a central assumption is that the sweet taste is actually perceived as rewarding. For individuals that do not like sweet taste, there is no reason to believe that repeated exposure to odor–sucrose mixtures would make the odors more pleasant. High interindividual variability in sweet-liking has therefore been proposed to explain the heterogeneity of previous results (Yeomans et al. 2006). Contrary to expectation, our study did not support the notion that sweet-liking modulates the amplifying effect of sweet-pairing on odor pleasantness. In subsequent exploratory analyses, we tested whether sweet-liking and the increase in pleasantness of sweet-paired odors were positively correlated. We found no evidence for such a relationship and a subsequent equivalence test confirmed that the hypothesized positive correlation was indeed weak at most. Taken together, our study did not provide any support for the proposed effect of associative learning on odor pleasantness.

The amplifying effects on pleasantness that arise due to learned associations between the post-ingestive consequences of eating and the food’s odor component might be sensitive to the metabolic state of the consumer at the point of testing (Gibson et al. 1995; Gibson and Wardle 2001). For example, people that have learned to associate an odor with a high intake of sucrose might evaluate that odor more favorably during hunger than during satiety. However, in the current study, we only instructed our participants to abstain from eating during the last hour before testing. The assumption hereby was that the amount of sucrose in the chewing gum was small enough to only have minor post-ingestive consequences. This means that we expected odor pleasantness to increase due to the synchronized sensory stimulation in itself, a learning process that is thought to be less dependent on hunger state (Mobini et al. 2007). Moreover, it is worth noting that our participants were instructed to chew the gums before their meals. While the aim of this instruction was to enhance the perceived reward value of sucrose, this procedure may at the same time have induced a preparatory physiological response of the digestive system (Smeets et al. 2010). Without an accompanying provision of calories, chewing might have elicited a negative emotional state with the potential to counteract any increase in liking for the odor. Previous studies have indicated that such preparatory responses can indeed occur as a result of chewing alone (Helman 1988). However, these effects likely require longer chewing sessions than what our participants completed (Teff 2010). It is therefore unlikely that physiological responses of the digestive system fully explain the absence of associative learning effects. Future studies are, however, needed to explore the role of hunger in this process.

Previous studies have shown that perceived odor intensity can be enhanced by taste, but only if the odor–taste combination is familiar (Green et al. 2012). Green and colleagues (2012) speculated that this effect might serve to enhance the salience of familiar nutritious foods. Given that odors provide the unique sensory profile necessary for object identification, while tastes provide information about macronutrient content, it seems likely that the function of odor enhancement by sweet taste is to strengthen the associative link between the food item and its nutritive value. To the best of our knowledge, this study was the first to directly test whether such effects on perceived odor intensity can be experimentally induced through exposure with sucrose. We found no evidence that this was the case, as the (non-significant) increase in odor enhancement by taste between the sessions did not vary depending on whether the odor had been presented with or without sucrose during the Exposure Phase. Results from an exploratory equivalence test further revealed that changes in odor intensity enhancement for sweet-paired odors were either absent or small in magnitude. Future studies assessing the potential effect of associative learning on odor intensity should consider that even if the effect exists, it likely needs a powerful experimental setup to be detectable.

We found no evidence that odor referral to the mouth varied depending on whether the odor had been presented with or without sucrose during the Exposure Phase. It has been known for over a century that, although the olfactory receptors are located in the nasal cavity, people frequently perceive the odor component of flavor in the mouth (Hollingworth and Poffenberger 1917). This illusion has been interpreted as a demonstration of the brain’s ability to create unified flavor percepts from odors and tastes (Stevenson 2014) and has been suggested to be the reason why people erroneously attribute olfactory content to the gustatory modality (vanilla yogurt is said to taste, not smell, like vanilla). Previous studies have shown that the perceived location of retronasal odors shifts toward the mouth when presented together with taste, but only if the odor–taste combination is familiar (e.g., Lim and Johnson 2011). The reliance on familiarity suggests that associative learning might play a role in creating this illusion. Our non-significant results add to the literature by suggesting that if odor referral is modulated by how often the odor and taste have been perceived together in the past, this effect likely requires a longer Exposure Phase to be detectable.

Lastly, we expected that perceived odor sweetness would increase more after exposure with sucrose than after exposure without taste. This hypothesis was not supported. Moreover, an exploratory equivalence test further suggested that the increase in odor sweetness corresponded to significantly less than 10% of our rating scale. This indicates that associative learning effects on perceived odor sweetness were, for our experimental setup, weak at most. These results are inconsistent with past studies that in general have reported large effects of exposure with sucrose on perceived odor sweetness (Stevenson et al. 1995, 1998, 2000a, 2000b; Stevenson and Case 2003; Prescott et al. 2004; Prescott and Murphy 2009; Yeomans et al. 2009; Stevenson and Mahmut 2011a, 2011b; Privitera et al. 2012; Yeomans and Boakes 2016). One possibility is that this discrepancy is related to differences in stimulus material. While we carefully piloted the target odors to ensure that they satisfied both the sensory (e.g., tasteless, clearly perceivable but not unpleasantly strong, moderately pleasant) and semantic (moderately familiar but not identifiable) requirements that have been suggested to facilitate odor–taste interactions, the basil and orange flower odorants have never been used in an exposure program before. We cannot rule out that these odors might have been more resistant to sweet-pairing than the odors that have been used previously. Odors that have been used in the past include water chestnut, lychee, oolong tea, raisin, and tea.

To formally assess the generalizability of the effect of sweet-pairing on odor sweetness, a broader stimulus set would be needed with a large number of target odorants. Such an approach would allow for statistical evaluation of between-odorant variability in the same way as testing multiple participants several times allows for evaluation of between-participant variability when making inferences at the population level (Yarkoni 2019). Given that such a diversity of stimulus material would require extensive and prolonged investments from any individual research group, coordinated efforts to promote team science would be highly desirable.

In addition to relying on a very small number of previously untested target odors, another possible limitation of our study that is worth highlighting is that we used chewing gums to present the stimuli during the Exposure Phase and liquids during the testing sessions. Previous studies have instead used liquids with dissolved odorants and tastants both for exposure and testing. We chose to use gums so that our participants could be exposed to the stimuli several times a day without having to visit the lab, and without having to handle liquid stimuli in their homes. Chewing gums can easily be carried everywhere and do not require careful storing to prevent flavor loss. However, this approach has at least two weaknesses that need to be acknowledged. First, we could not control the external conditions during the actual exposures. While we instructed our participants to chew in a quiet place and pay full attention to the flavors, we do not know how strictly this instruction was followed. Second, our stimulus presentation matrix required participants to generalize what might have been encoded through one presentation mode (chewing gum) to another presentation mode (liquid) during retrieval. In real life, our perceptual systems seem to handle such inconsistencies rather seamlessly. Lemon flavor is, for example, perceived and evaluated in a similar fashion in juice, fruit, and chewing gums. However, we cannot rule out that potential effects would have emerged if the stimulus presentations during the testing sessions and Exposure Phase had been the same.

One final possible explanation for the lack of increase in odor sweetness is that associative learning effects might be weaker than previous studies have suggested. In that case, our sample size would not have been large enough to detect the true effect. This explanation would be consistent with large-scale replication studies from other sub-fields within psychology, which on average have produced effect sizes that are about half as strong as those reported in the original studies (Camerer et al. 2018; Klein et al. 2018; Open Science Collaboration 2015). Based on everyday eating experience, small effects seem indeed plausible: the observation that many odors that exclusively appear in desserts (e.g., vanilla) have sweet smells clearly suggests that repeated exposure with taste can alter how the odor is subsequently processed. Yet, while most would probably agree that vanilla smells sweet, we seldomly experience that odor sweetness changes noticeably through a few exposures. In fact, many non-sweet food odors, such as chicken, do regularly appear together with sweet taste (e.g., honey roasted chicken) without becoming sweet-smelling. While our study thus does not challenge the validity of previous work due to the methodological discrepancies mentioned above, a preregistered and independent direct replication of a prior study that has reported a large effect would be highly informative. Such a study would be useful to confirm that associative learning reliably can affect odor sweetness under specific conditions. Also, it would act as a stepping stone for more systematic investigations of the types of exposure programs that are best suited to detect the potential effect.

In conclusion, the aim of this study was to disentangle associative learning effects from effects of exposure without taste on four food-relevant outcomes that have been suggested to rely on past experience: odor sweetness, odor pleasantness, odor intensity enhancement by taste, and odor referral to the mouth. Our hypotheses were assessed using a large sample compared with other studies in the field and data were analyzed according to a preregistered analysis plan. Contrary to expectation, our results did not provide any evidence for associative learning effects on any of the outcomes. To our best knowledge, this is the first time that odor intensity enhancement by taste and odor referral to the mouth has been assessed in an exposure-based learning study. Moreover, as previous attempts to amplify odor pleasantness by exposure with sucrose have yielded heterogeneous results, the absence of associative learning effects on pleasantness in our study does not deviate much from the existing literature. Future studies should consider the possibility that if these three phenomena rely on associative learning, the effects might be fragile and not so easy to induce experimentally. However, our observed null effect on odor sweetness does contrast with several previous reports. This discrepancy might indicate that the acquisition of learned associations between odor and taste during food consumption is dependent on the context that the exposure takes place in, or that some odors might be more resistant to sweet-pairing than others. Alternatively, the observed lack of increase in odor sweetness might indicate that the true effect is smaller than previously thought. A high-powered direct replication of a study with significant results would be highly informative to assess the reliability of the effect of sweet-pairing on odor sweetness. If the phenomenon is replicable, further exploration of potential influencing factors and a conscious effort to create comparable setups between different research groups would be desirable to increase our understanding of the specific conditions under which such odor–taste associations are formed. Taken together, these efforts will have the potential to transform our understanding of how food perception and eating behavior develop over time, and thus provide important insights into the perceptual basis of human food choice.
